# Rheumatoid arthritis and the risk of ischaemic stroke after diagnosis of atrial fibrillation: a Norwegian nationwide register study

**DOI:** 10.1093/rheumatology/keae458

**Published:** 2024-08-22

**Authors:** Anne M Kerola, Eirik Ikdahl, Ingrid Engebretsen, Christoffer Bugge, Anne Grete Semb

**Affiliations:** Department of Rheumatology, Inflammation Center, Helsinki University Hospital and University of Helsinki, Helsinki, Finland; Department of Internal Medicine, Päijät-Häme Central Hospital, Lahti, Finland; Department of Rheumatology, REMEDY-Centre for Treatment of Rheumatic and Musculoskeletal Diseases, Diakonhjemmet Hospital, Oslo, Norway; Department of Health and Life science, Oslo Economics, Oslo, Norway; Department of Health and Life science, Oslo Economics, Oslo, Norway; Preventive Cardio-Rheuma Clinic, Section for Research and Innovation, REMEDY-Centre for Treatment of Rheumatic and Musculoskeletal Diseases, Diakonhjemmet Hospital, Oslo, Norway

**Keywords:** RA, atrial fibrillation, ischaemic stroke, registry study, competing risks

## Abstract

**Objectives:**

RA patients have an increased risk for cardiovascular diseases, including atrial fibrillation (AF), but the impact of RA on ischaemic stroke risk in the context of AF remains unknown. We explored whether the risk of ischaemic stroke after diagnosis of AF is further increased among patients with RA compared with non-RA patients.

**Methods:**

In the nationwide Norwegian Cardio-Rheuma Register, we evaluated cumulative incidence and hazard rate of ischaemic stroke after the first AF diagnosis (2750 individuals with RA and 158 879 without RA between 2010 and 2017) by using a competing risk model with a 3-month delayed entry.

**Results:**

The 5-year unadjusted cumulative incidence of ischaemic stroke was 7.3% (95% CI: 5.9–8.7%) for patients with RA and 5.0% (95% CI: 4.9–5.2%) for patients without RA. Unadjusted univariate analyses indicated that AF patients with RA had a HR of 1.36 (95% CI: 1.13, 1.62) for ischaemic stroke compared with those without RA. Sex- and age-adjusted HR for ischaemic stroke in RA patients with AF was 1.25 (95% CI: 1.05, 1.50), and the effect size remained unchanged after adjustment for diabetes, hypertension, atherosclerotic cardiovascular disease and oral anticoagulant (OAC) treatment. RA patients were less likely to receive OAC treatment than non-RA patients (adjusted odds ratio 0.88, 95% CI: 0.80, 0.97).

**Conclusion:**

RA patients diagnosed with AF are at a further increased risk for stroke compared with non-RA patients with AF, and less likely to receive OAC treatment, emphasizing the need to improve stroke prevention in AF patients with RA.

Rheumatology key messages

- RA is an additional risk factor for ischaemic stroke among patients with atrial fibrillation.
- RA patients with AF are less likely to receive oral anticoagulation than non-RA patients.
- Stroke prevention in AF patients with RA warrants cardiovascular risk factor control and increasing OAC coverage.

## Introduction

Atrial fibrillation (AF) is the most frequent sustained arrhythmia [1], resulting in a 2-fold increased mortality and a 5-fold increased stroke risk [2]. However, this risk increase is not homogeneous across AF patients but depends on the presence of stroke risk factors/modifiers, such as sex, age and the presence of other CVDs and their risk factors [3]. In AF patients with stroke risk factors, oral anticoagulant (OAC) therapy can reduce the risk of ischaemic stroke effectively [3]. CHA2DS2-VASc score is used to identify patients at a high risk of stroke who are likely to benefit from OAC treatment [3]. However, not all factors that increase stroke risk in patients with AF, such as sleep apnoea and renal failure, are included in this score [4].

RA is an inflammatory joint disease associated with an increased risk for several cardiovascular diseases (CVD) and cardiovascular mortality [5, 6]. The increased risk of atherosclerosis results from the combined effects of traditional CVD risk factors and systemic inflammation [6]. Interestingly, RA is also known to increase the risk of AF to ∼1.4-fold [7–9]. Also, evidence points towards an ∼1.4-fold risk of ischaemic stroke among RA patients compared with non-RA controls [7]. However, it has not been elucidated whether RA increases the risk of ischaemic stroke in the specific population of patients with AF, in whom ischaemic stroke especially of cardioembolic origin can be prevented with OAC treatment.

Regarding stroke prevention in patients with AF, our international audit, SURF-RA, showed that a considerable proportion of patients with RA and AF with an indication to OAC therapy may not receive this treatment [10], a phenomenon also common in the general population [11]. It remains unknown whether RA patients with AF are less likely to receive OAC treatment compared with non-RA patients. Moreover, information on the impact of RA on the magnitude of ischaemic stroke risk among patients with AF may offer additional support in decision-making regarding OAC treatment in this vulnerable patient population.

Using nationwide registry data comprising the entire Norwegian population, we set out to assess if the risk of ischaemic stroke after diagnosis of AF among patients is further increased among AF patients with RA compared with those without RA. We also assessed whether RA patients with AF are less likely to receive OAC treatment for stroke prevention compared with non-RA patients.

## Methods

### Study design and data sources

We conducted a registry-based retrospective cohort study on the risk of ischaemic stroke among AF patients with and without RA using the Norwegian Cardio-Rheuma Register (NCRR) encompassing the entire adult population of Norway (4.7 million individuals aged 18 years and above). The follow-up started 3 months after the first AF diagnosis (see *Statistical analyses* for details). Data from the Norwegian Patient Registry (NPR), the Norwegian Prescription Database (NorPD), the Norwegian Cause of Death registry (NCDR) and Statistics Norway spanning the period from 1 January 2008 to 31 December 2017 were linked using individual personal identification numbers. The NPR holds data on all treatments in publicly funded hospitals in Norway, including in- and outpatient care. As publicly funded hospitals in Norway are responsible for most of the treatment in Norway, the data from NPR covers the vast majority of Norwegian patients diagnosed with AF. Each hospital encounter is recorded with a primary and possibly secondary diagnosis using the International Classification of Diseases, Tenth Revision (ICD-10). The NorPD holds data on all pharmacy-dispensed prescription drugs (reimbursed or not). Each drug prescription is recorded with a reimbursement code, which is equivalent to an ICD-10 code in specialist care and ICPC-2 (International Classification of Primary Care, second edition) code in primary care, and a unique patient identifier. All pharmacies in Norway are required by law to send electronic data to NorPD and the registry is regarded to be close to complete [12]. The NCDR dates back to 1951 and holds information on time and cause of death for all deaths in Norway, regardless of whether the deceased are Norwegian citizens, or if the death occur outside of Norway (for Norwegian citizens) [13]. Patient characteristics were obtained from Statistics Norway.

### Identification and classification of patients

Cases were identified and classified based on their encounters with hospitals and use of prescription drugs utilizing primary and secondary diagnosis codes in NPR (ICD-10) and reimbursement codes in NorPD (ICD-10 and ICPC-2).

#### Study population

**Figure 1.**
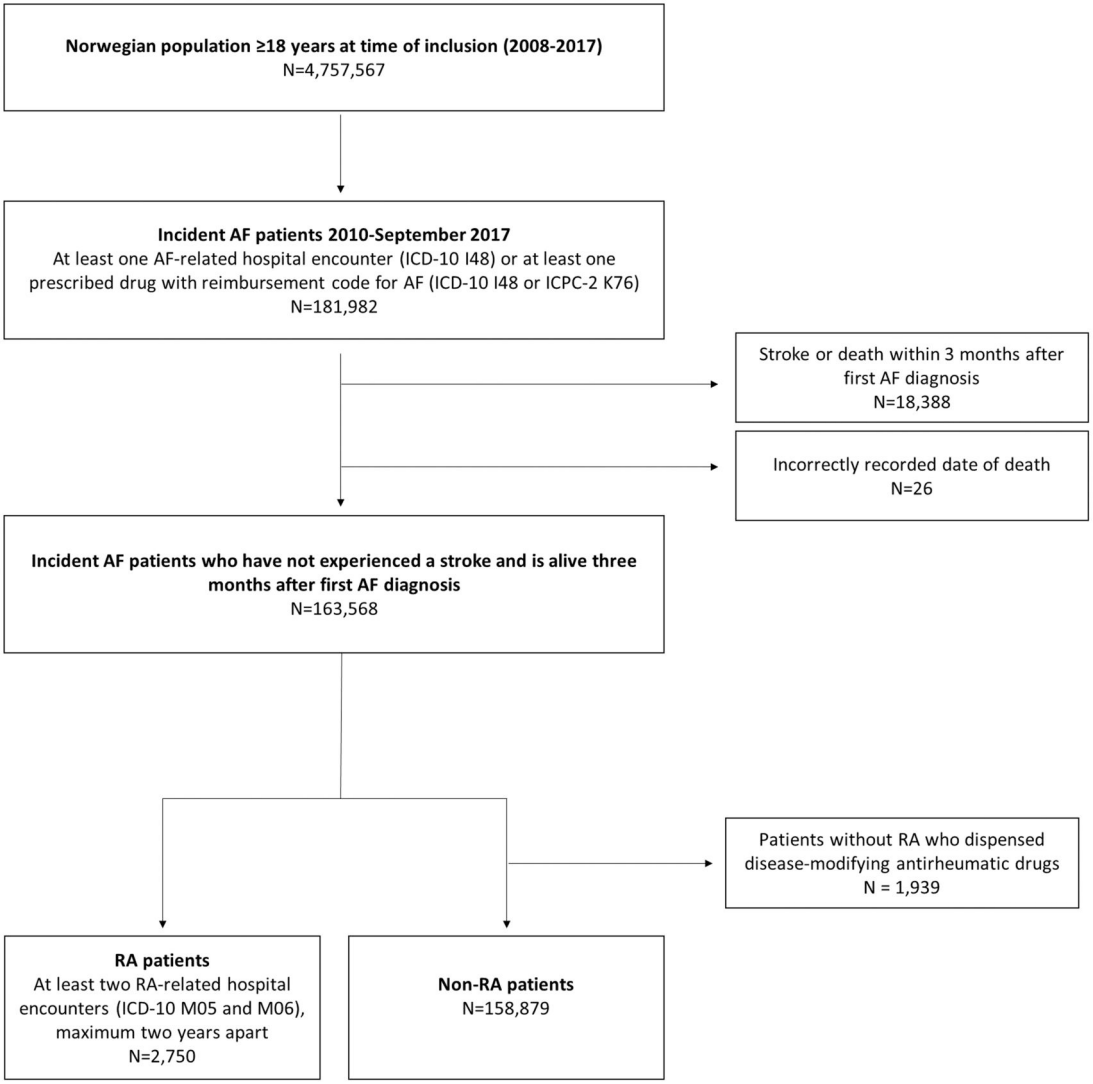
Study population

Newly-diagnosed AF patients between 2010 and 2017 were included in the study (*n* = 163 595) (Fig. 1). Patients with AF were identified as patients with at least one AF or atrial flutter-related hospital encounter (ICD-10 code I48) or at least one prescribed drug recorded with a reimbursement code for AF (ICPC-2 K78 or ICD-10 I48) during the period 2010–2017. To identify time of first AF diagnosis, we used a wash-out period of 2008 and 2009 by assuming that patients with no AF-related treatment episodes or reimbursed prescriptions during these years were incident cases. First AF diagnosis was defined as whichever came first, hospital encounter or prescription.

#### Exposure: RA

Patients were defined as having RA if they had at least two RA-related hospital encounters (ICD-10 M05 or M06), maximum two years apart [14]. We only identified RA patients who fulfilled these criteria before or at the same time as their first AF diagnosis. If a patient was diagnosed with RA after the detection of AF, they stayed in the non-RA group. Patients without RA who received disease-modifying anti-rheumatic drugs (*n* = 1939) were excluded from the study to ensure that the controls (persons without RA) did not have another rheumatic disease.

#### Primary end point: ischaemic stroke

The primary end point was ischaemic stroke and was identified using hospital encounters recorded with ICD-10 I63 in the NPR. Secondary endpoints included two subgroups of ischaemic strokes: cerebral infarction due to embolism of cerebral arteries (ICD-10 I63.4) and cerebral infarction due to other or unspecified causes (ICD-10 I63.0-I63.3, I63.5-I63.9).

#### Baseline comorbidities

We classified patients based on their comorbidities, including diabetes (ICD-10 E10-E14), hypertension (ICD-10 I10), atherosclerotic CVD (ischaemic heart disease or peripheral artery disease) (ICD-10 I70, I21-25), congestive heart failure (ICD-10 I11.0, I50) and previous stroke or transient ischaemic attack (TIA) (ICD-10 I63, I64, I66, G45, G46). Patients were classified as having the comorbidity if they had at least one hospital encounter recorded with the above-mentioned ICD-10 code. In addition, patients were classified as having diabetes or hypertension if they had at least two prescriptions registered with one of the relevant reimbursement codes (ICD-10 E10-E14 or ICPC-2 T89-T90 for diabetes or ICD-10 I10 or ICPC-2 K86-K87 for hypertension).

For all comorbidities except stroke or TIA, we classified patients as having the disease if at least one of the diagnostic/reimbursement codes were recorded in the same month or prior to first AF diagnosis. For stroke and TIA, the diagnostic code was required to have been recorded prior to the month of first AF diagnosis.

#### OAC treatment

Treatment with oral anticoagulants (OAC) were defined as having had at least one dispensed prescription of OAC in NorPD (ATC B01AA, B01AC, B01AE or B01AF) within 3 months of the first recorded AF diagnosis.

#### Anti-rheumatic treatment

We define the patient to be treated with anti-rheumatic medication (DMARDs) if they had at least one dispensed prescription of a synthetic DMARD in NorPD (ATC-codes L01BA01, L04AX03, A07EC01, L04AX01, P01BA02, M01CB, L04AA13, L01AA01, L04AD01) or of a biological DMARD (ATC-codes L01XC02, L04AA24, L04AB01, L04AB02, L04AB04, L04AB0, L04AB06, L04AC03, L04AC05, L04AC07, L04AC10) between first AF diagnosis and 3 months after first diagnosis of AF.

#### CHA_2_DS_2_-VASc score

Risk of ischaemic stroke was described using the CHA_2_DS_2_-VASc score. In calculation of CHA_2_DS_2_-VASc score, the presence of congestive heart failure, hypertension, diabetes, vascular disease, age 65–74 years and female sex yields one point each, and age ≥ 75 years and a history of stroke yield two points each, with a maximum score of nine [10]. Age was defined at first AF diagnosis. Vascular disease required at least one previous myocardial infarction or peripheral arterial disease contact (ICD-10 I21, I22, I70) in the NPR.

### Statistical analyses

Cumulative incidence and hazard rates of ischaemic stroke were analysed with a 3-month delayed entry model, in which calculating time at risk and events started from 3 months after first AF diagnosis (start of follow-up period). We measured time in days from entry to end of follow-up [i.e. ischaemic stroke (ICD-10 code I63 from the NPR), death (from the NCDR) or end of data period (31 December 2017), whichever came first]. A 3-month delayed entry model was chosen because we wanted to exclude patients that were diagnosed with AF and ischaemic stroke simultaneously, without a possibility for stroke prevention with OAC treatment. In addition, this approach allowed us to adjust our analyses with OAC use (defined as an OAC purchase during the first 3 months after AF diagnosis).

We calculated the unadjusted cumulative incidence of ischaemic stroke and 95% CI for AF patients with and without RA at 1, 3 and 5 years. Hazard rates were estimated using a competing risk regression models (Fine-Gray regression model) with time-invariant covariates [15]. Death was considered a competing risk to prevent the overestimation of ischaemic stroke occurrences. The models included the following covariates for each individual patient: RA, sex, age, comorbidities prior to the initial AF diagnosis, whether the patient received OAC treatment and whether the patient received anti-rheumatic treatment. Adjusted subgroups analyses were performed for all covariates.

We estimated the odds ratio of receiving OAC treatment for RA patients compared with non-RA patients with logistic regression with adjustment for CHA_2_DS_2_-VASc score. Furthermore, we performed a sensitivity analysis with adjustment for age, sex and comorbidities instead of CHA_2_DS_2_-VASc score.

In a subgroup analysis of RA patients, we assessed the effect of using anti-rheumatic treatment on the hazard ratio (HR) of ischaemic stroke, with adjustments for age, sex, comorbidities and OAC treatment.

All analyses were conducted using R version 4.1.2 (2021).

### Ethics approval

The study was approved by the Norwegian General Data Protection Regulation (16/00482–11/CDG), the South East Health Authority Ethical Committee (2016/588) and the Data Protection Officers at Oslo University Hospital (2016/924) and Diakonhjemmet Hospital (7/12–2019). The NCRR comprises routinely recorded administrative data, and no written consent from study subjects was required.

## Results

In total, 161 629 patients were identified as having incident AF during 2010–2017, of which 2750 (1.7%) had RA. The median (IQR) follow-up time for patients with RA was 2.5 (3.4) years and 3.0 (3.75) years for patients without RA, with a maximum follow-up of 7.75 years.

RA patients were more often female and somewhat older compared non-RA patients (Table 1). Congestive heart failure, atherosclerotic CVD, hypertension, diabetes and stroke or TIA prior to first AF diagnosis were significantly more prevalent among AF patients with RA compared with AF patients without RA (*P* < 0.001). Mean CHA_2_DS_2_-VASc score was higher among AF patients with RA compared with non-RA patients. The distribution of CHA_2_DS_2_-VASc score is presented in Supplementary Table S1, available at *Rheumatology* online.

**Table 1.** Age, sex, comorbidities and medications among AF patients with and without RA

	AF and RA	AF without RA
	*n* = 2750	*n* = 158 879
Females, *n* (%)	1738 (63.2)	69 339 (43.6)
Age at first AF diagnosis, median (IQR)	76 (14)	73 (19)
Age groups, *n* (%)		
<60	217 (7.9)	29 059 (18.3)
60–64	220 (8.0)	14 556 (9.2)
65–69	373 (13.6)	21 083 (13.3)
70–74	470 (17.1)	22 284 (14.0)
75–79	511 (18.6)	21 851 (13.8)
80–84	523 (19.0)	21 480 (13.5)
85–89	321 (11.7)	17 563 (11.1)
90+	115 (4.2)	11 003 (6.9)
Comorbidities prior to first AF diagnosis, *n* (%)		
Congestive heart failure	497 (18.1)	23 020 (14.5)
Atherosclerotic CVD	897 (32.1)	41 257 (26.0)
Hypertension	1894 (68.9)	94 990 (59.0)
Diabetes	444 (16.2)	20 827 (13.1)
Stroke or TIA	222 (8.1)	10 314 (6.5)
CHA_2_DS_2_-VASc score, mean (SD)	3.40 (1.53)	2.76 (1.64)
OAC treatment 0–3 months after first AF diagnosis, *n* (%)	2125 (77.3)	119 808 (75.4)
DMARDs 0–3 months after first AF diagnosis, *n* (%)	1207 (43.9)	NA
Biological DMARDs	401 (14.6)	NA
Conventional synthetic DMARDs	1173 (42.7)	NA

### OAC treatment

When adjusting for CHA_2_DS_2_-VASc score, the estimated effect of having RA on the odds ratio (OR) of receiving OAC treatment during the 3-month period after AF diagnosis was 0.88 (95% CI: 0.80, 0.97). When adjusting for age, sex and comorbidities (including diabetes, hypertension and atherosclerotic CVD), the estimated effect was similar (OR: 0.88, 95% CI: 0.80, 0.97).

### Cumulative incidence of ischaemic stroke

During the follow-up, 121 (4.4%) of the AF patients with RA and 5463 (3.4%) of the AF patients without RA had an ischaemic stroke. The causes of the ischaemic strokes, as evaluated by ICD-10 coding, are shown in Supplementary Table S2, available at *Rheumatology* online. Cerebral infarction due to unspecified cause (I63.9) was the most frequently used ICD-10 code, followed by cerebral infarction due to embolism of cerebral arteries (I63.4). Mortality during the follow-up was high: a total of 803 (29.2%) RA patients and 34 121 (21.5%) controls died.

**Figure 2.**
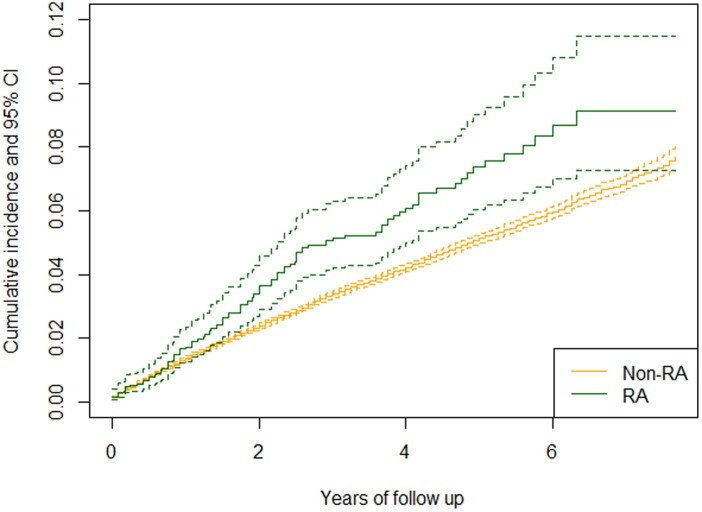
Unadjusted cumulative incidence of ischaemic stroke after AF diagnosis in patients with and without RA

After one year of follow-up, the unadjusted cumulative incidence of ischaemic stroke was 1.8% (95% CI: 1.2–2.3%) for patients with RA and 1.4% (1.3–1.4%) for patients without RA (Fig. 2). In longer follow-up, the cumulative incidence curves diverged: at three years, cumulative incidence of ischaemic stroke was 5.0% (95% CI: 4.0–6.0%) among RA patients and 3.3% (3.2–3.4%) among non-RA patients, and at five years 7.3% (5.9–8.7%) and 5.0% (4.9–5.2%), respectively. The cumulative incidence curves for ischaemic stroke due to embolism of cerebral arteries and other or unspecified causes are shown in Supplementary Fig. S1A and B, available at *Rheumatology* online.

**Figure 3.**
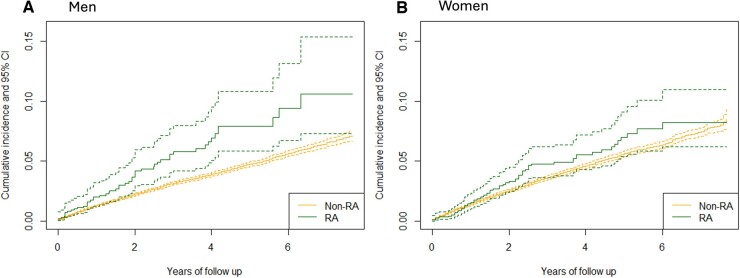
(A and B) Cumulative incidence of ischaemic stroke in RA and non-RA cases after AF diagnosis in (A) men (*N*: RA = 1012, non-RA: 89 450) and (B) women (*N*: RA = 1738, non-RA: 69 339)

Looking at subgroups by sex, the cumulative incidence of ischaemic stroke was higher among men with RA compared with men without RA (Fig. 3A). The 1-year cumulative incidence was 2.0% (95% CI: 1.1–2.9%) in men with RA *vs* 1.3% (1.2–1.4%) in men without RA, at 3 years 5.4% (4.7–7.1%) *vs* 3.1% (3.0–3.2%), and at 5 years 7.6% (5.4–9.9%) *vs* 4.7% (4.5–4.8%), respectively. In women, the 95% CIs were largely overlapping (Fig. 3B). The 1-year cumulative incidence was 1.5% (95% CI: 0.9–2.2%) in women with RA *vs* 1.4% (1.3–1.5%) in women without RA, at 3 years 4.6% (3.4–5.8%) *vs* 3.5% (3.4–3.7%), and at 5 years 6.8% (5.0–8.5%) *vs* 5.5% (5.2–5.8%). The cumulative incidence curves for ischaemic stroke in RA patients compared with non-RA patients in several patient subgroups are presented in Supplementary Fig. S2, available at *Rheumatology* online.

### Relative risk of ischaemic stroke

We developed multiple regression models by adding covariates to investigate the influence of having RA on the risk of ischaemic stroke after first AF diagnosis (Table 2). In the unadjusted univariate analysis, the AF patients with RA had a HR of 1.36 (95% CI: 1.13, 1.62) for ischaemic stroke, compared with those without RA (Model 1). Upon adjusting for sex and age (Model 2), AF patients with RA had an estimated HR of 1.25 (95% CI: 1.05, 1.50). After adding diabetes, hypertension and atherosclerotic CVD (Model 3) and OAC treatment (Model 4) to the model, the effect of having RA on risk of ischaemic stroke remained similar. The risk for ischaemic stroke due to embolism of cerebral arteries (ICD-10 I63.4) was not significantly elevated, the age- and sex-adjusted HR being 1.12 (95% CI: 0.81, 1.55). However, the HR for other and unspecified ischaemic strokes (I63.0-I63.3, I63.5-I63.9) was 1.32 (95% CI: 1.06, 1.64).

**Table 2.** Fine-Gray regression results—hazard ratio of ischaemic stroke among AF patients

	Hazard ratio [95% CI]			
	Model 1	Model 2	Model 3	Model 4
RA	1.36	1.25	1.22	1.22
	[1.13, 1.62]	[1.05, 1.50]	[1.02, 1.46]	[1.02, 1.46]
Age at AF diagnosis (reference: 0–59 years)				
60–64		1.96	1.79	1.80
		[1.69, 2.28]	[1.54, 2.07]	[1.55, 2.10]
65–69		2.38	2.11	2.13
		[2.09, 2.71]	[1.84, 2.41]	[1.86, 2.44]
70–74		3.04	2.64	2.68
		[2.68, 3.45]	[2.33, 3.00]	[2.35, 3.06]
75–79		3.72	3.18	3.23
		[3.29, 4.20]	[2.81, 3.61]	[2.84, 3.68]
80–84		5.20	4.44	4.50
		[4.61, 5.86]	[3.92, 5.02]	[3.96, 5.11]
85–89		6.01	5.20	5.27
		[5.38, 6.86]	[4.58, 5.89]	[4.63, 5.99]
90+		6.93	6.01	6.07
		[6.07, 7.91]	[5.24, 6.88]	[5.28, 6.97]
Female		0.91	0.93	0.92
		[0.86, 0.96]	[0.88, 0.98]	[0.87, 0.97]
Diabetes			1.27	1.27
			[1.18, 1.36]	[1.18, 1.36]
Hypertension			1.36	1.37
			[1.28, 1.45]	[1.29, 1.46]
Atherosclerotic CVD			1.13	1.13
			[1.06, 1.19]	[1.07, 1.20]
OAC treatment				0.95
				[0.80, 1.02]

**Figure 4.**
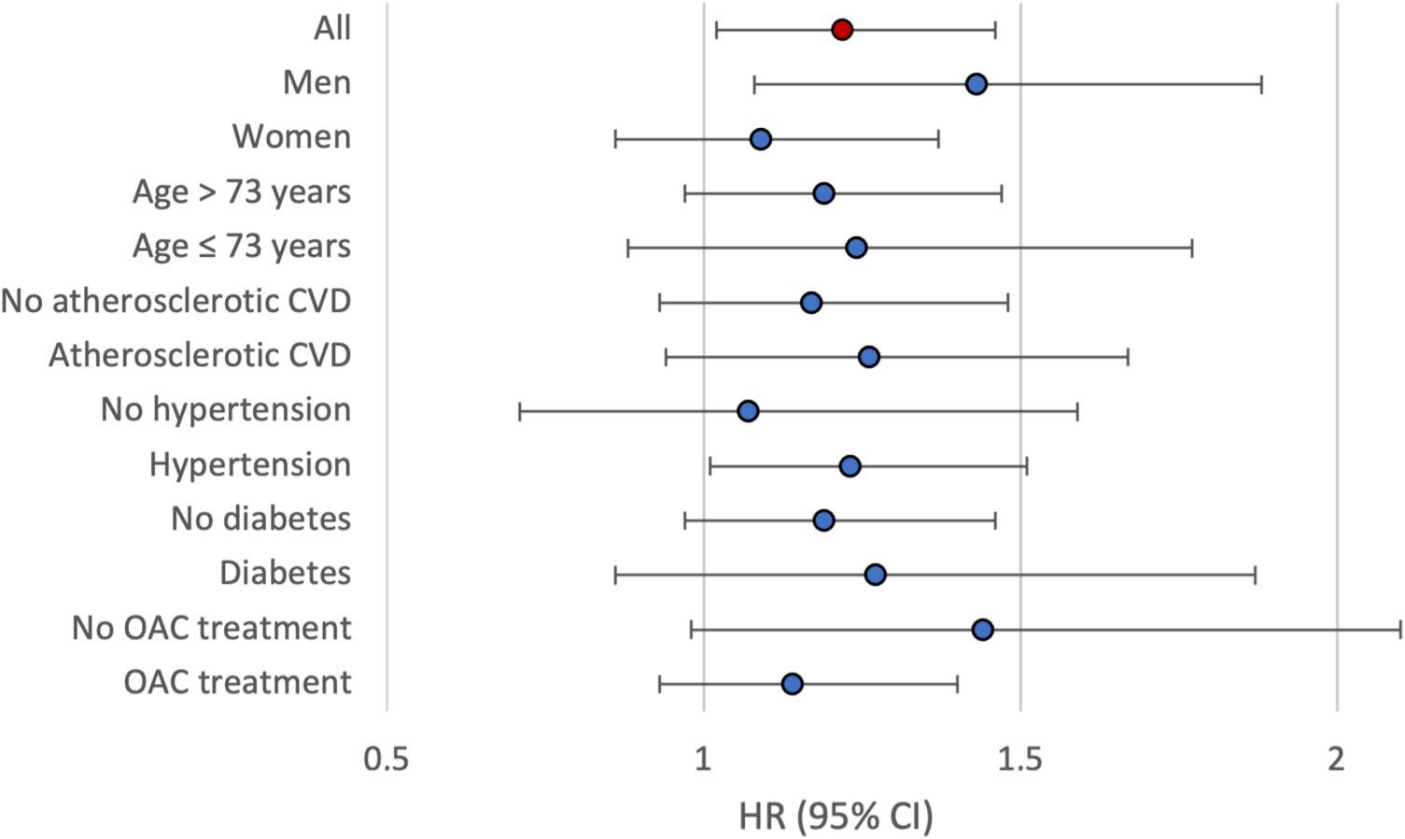
Adjusted estimates of the effect of RA on the risk of ischaemic stroke among patient subgroups

In subgroup analyses, adjusted HR point estimates of the effect of RA on risk of ischaemic stroke varied slightly between subgroups (Fig. 4). However, many subgroup analyses failed to reach the level of statistical significance. Among men, the presence of RA was associated with a HR of 1.43 (95% CI: 1.08, 1.88, *P* = 0.01) for ischaemic stroke, while in women, no statistically significant difference was found (HR 1.09; 95% CI: 0.86, 1.37; *P* = 0.48). Among patients with hypertension the HR was 1.23 (95% CI: 1.01, 1.51, *P* = 0.04), with no statistically significant difference in patients without hypertension. Finally, among patients not receiving OAC treatment, RA patients had a HR of 1.44 for ischaemic stroke compared with patients without RA (95% CI: 0.98, 2.10), but the effect was not significant at the 5% confidence level (*P*-value 0.06).

### Impact of DMARD treatment

We assessed the risk of ischaemic stroke among RA patients with AF using anti-rheumatic treatment compared with non-users. In univariate analysis, the HR of ischaemic stroke among RA patients using anti-rheumatic treatment was 0.74 (95% CI: 0.52, 1.07). When adjusting for age, sex and comorbidities, the estimated HR was 0.85 (95% CI: 0.59, 1.24). When adjusting for age, sex, comorbidities and OAC treatment, the estimated effect was 0.86 (95% CI: 0.59, 1.25).

## Discussion

In this nationwide registry study, we showed that the presence of RA is associated with an increased risk of ischaemic stroke after AF diagnosis during a long follow-up. Previously, it has been shown that RA patients in general have an increased risk of stroke compared with non-RA controls: a meta-analysis of observational studies yielded a sex- and age-adjusted risk ratio of 1.38 (95% CI: 1.29, 1.48) for ischaemic or haemorrhagic stroke, and the estimate remained similar after adjustment for at least one traditional risk factor [9]. To our knowledge, this increased stroke risk has not been previously shown to persist in the context of AF. Our study prompts preventive measures such as meticulous cardiovascular risk factor control among patients with RA and AF, and raises the question whether the presence of RA should be taken into account when considering OAC treatment for AF patients.

Many potential explanations for our main finding merit discussion. One might argue that the overrepresentation of traditional CVD risk factors including diabetes, hypertension and obesity, as well as overrepresentation of atherosclerotic CVD in RA underlies the increased risk of ischaemic stroke, even in the context of AF [16]. However, our main finding remained similar after adjustment for diabetes, hypertension, atherosclerotic CVD, although residual confounding is nevertheless possible. Certain other stroke risk factors are also overrepresented among RA patients, such as smoking, carotid atherosclerosis and vulnerable carotid plaques [17–19]. Besides acceleration of atherosclerosis, systemic inflammation in active RA may exert pro-thrombotic effects such as endothelial injury and hypercoagulability [20]. It has also been proposed that RA-related inflammation leads to left atrial myopathy, which in turn increases the risk of thromboembolic stroke [21]. Of RA medications, the risk of CVD events may be increased by glucocorticoid use [22], which was rather common among RA patients in this cohort [23].

The stroke subtype most commonly associated with AF is cardioembolic stroke, which often presents with particularly severe symptoms and leads to the highest rates of mortality and permanent disability [24]. Unfortunately, we were not able to reliably identify cardioembolic events based on ICD-10 codes. Most of the ischaemic stroke events were recorded with the ICD-10 code I63.9 for unspecified cerebral infarction. As a secondary analysis, we assessed the risk of ischaemic stroke due to embolism of cerebral arteries (I63.4) and, perhaps due to lack of power, were unable to detect a difference between RA patients and controls. Future studies with data on the clinical and imaging features of the cerebral infarction are warranted.

We did not find any difference in the risk of ischaemic stroke between RA patients with and without DMARD treatment. These results should be interpreted with caution, since we were unable to adjust for disease activity, and our analyses were also limited by the low number of ischaemic strokes among RA patients with DMARDs. Of note, the use of Janus kinase inhibitors, a group of DMARDs that have been linked to an increased risk of venous thromboembolism and major adverse cardiovascular events [25], was very rare among our RA cohort during the study period [23].

RA patients were less likely to receive OAC treatment during the 3-month period after AF diagnosis compared with patients without RA, even after adjustment for CHA_2_DS_2_-VASc score. This may partly explain the increased risk of ischaemic stroke in RA patients with AF. Underuse of OACs in AF patients at a high stroke risk is a common problem in both patients with and without RA, and contributes disproportionately to large strokes requiring endovascular therapy [10, 26, 27]. According to our international audit among RA patients in 17 countries, up to 35% of AF patients with an indication to OAC treatment were not treated [10]. Speculatively, concerns of polypharmacy and use of non-steroidal anti-inflammatory drugs or glucocorticoids may decrease the propensity of physicians to prescribe OAC therapy to patients with RA. The reasons for the gap in OAC treatment among RA patients warrant further qualitative studies.

The performance of CHA_2_DS_2_-VASc score in patients with RA has not been studied in the context of AF, but only in predicting the risk of mortality, hospitalization and ischaemic stroke in RA patients without AF [28, 29]. Future studies are prompted to elucidate whether adding RA as a factor to CHA_2_DS_2_-VASc score would improve the score’s predictive value for this patient group.

When exploring our results by sex, men with RA had a higher risk of ischaemic stroke compared with men without RA, but in women, we detected no statistically significant differences. The reasons for this finding are unclear. According to a review, the influence of sex on CVD risk among RA patients is controversial, but men with RA may be at a higher risk of pericarditis, ischaemic heart disease, heart failure with reduced ejection fraction and cardiovascular mortality, compared with women with RA [30].

We used a 3-month delayed entry model to study long-term ischaemic stroke risk for two reasons. Firstly, we wanted to exclude patients who were diagnosed with AF and ischaemic stroke during the same admission, since prevention of ischaemic stroke with OAC treatment could have not been implemented in these patients. Secondly, 3 months was a reasonable time frame to identify OAC treatment after AF diagnosis, allowing us to adjust our analyses for OAC treatment.

A strength to our study is its nationwide design, with good generalizability to countries with similar healthcare systems and similar background CVD risk. Some features of registry studies relying on diagnostic codes and drug purchases must be taken into account when interpreting our results. We used a wash-out period of two years to identify the majority of patients with prevalent CVD risk factors and AF. Nevertheless, some cases may still remain unidentified, especially if they are not seen by a doctor regularly and are not treated with pharmacotherapy. We lack data on smoking, obesity, blood pressure measurements, ECG documentations for AF, alcohol use and many risk factors for bleeding, which would ideally be adjusted for when exploring ischaemic stroke risk and probability of receiving OAC treatment. Whereas smoking is rather uncommon in Norway (in 2015, 13% of Norwegians aged 13–74 smoked daily [31]), obesity is not (52% of adult males and 38% of adult females were overweight in 2006–2008 in the population-based HUNT3 study [32]). Both smoking and obesity are risk factors for not only for AF and ischaemic stroke but also RA [6, 33], which may induce bias. We acknowledge a potential source of surveillance bias: since RA patients are often followed up regularly by internal medicine specialists and they are recommended to undergo cardiovascular risk assessment, asymptomatic AF and CVD risk factors could be detected earlier among RA patients compared with non-RA patients. However, this may only lead us to underestimate the RA-related excess risk of ischaemic stroke, and does not explain why RA patients with AF received less OAC treatment, because AF symptoms and AF burden should not impact the threshold to initiate OAC treatment.

In conclusion, our nationwide registry study provides evidence that the presence of RA is associated with an increased risk of ischaemic stroke in the context of AF, regardless of the presence of diabetes, hypertension and atherosclerotic CVD. In addition, RA patients with AF were less likely to receive OAC treatment than non-RA patients, which may partly explain our main finding. Future studies are warranted to explore the effect of implementation of RA as a factor in CHA_2_DS_2_-VASc score, and whether increasing OAC coverage among AF patients with RA would aid in preventing ischaemic strokes.

## Supplementary Material

keae458_Supplementary_Data

## Data Availability

Due to legal and privacy considerations, the research data that support the findings of this study will not be made available to the public.
