# Supplementary material for: Rheumatoid arthritis and the risk of ischaemic stroke after diagnosis of atrial fibrillation: a Norwegian nationwide register study
Source: Rheumatology (Oxford). 2024 Aug 22;63(11):2997–3005. doi: 10.1093/rheumatology/keae458 (PMC11534115; doi:10.1093/rheumatology/keae458)

Supplementary material

Table S1. Distribution of CHA2DS2-VASc score.

CHA2DS2-VASc score	Number of patients (%)	
	AF and RA	AF and non-RA
0-1	306 (11.1%)	39 199 (24.7%)
2-3	1114 (40.5%	66 820 (42.1%)
4-5	1109 (40.3%)	44 943 (28.3%)
6-7	207 (7.5%)	7 478 (4.7%)
8-9	14 (0.5%)	439 (0.3%)

Table S2. Distribution of cerebral infarctions by their cause according to ICD-10 coding.

		Number of events	
**ICD-10**	**Cerebral infarction due to:**	**RA**	**Non-RA**
I63.0	Thrombosis of precerebral arteries	3	111
I63.1	Embolism of precerebral arteries	7	111
I63.2	Unspecified occlusion or stenosis of precerebral arteries	3	104
I63.3	Thrombosis of cerebral arteries	14	574
I63.4	Embolism of cerebral arteries	37	1838
I63.5	Unspecified occlusion or stenosis of cerebral arteries	5	369
I63.6	Cerebral venous thrombosis, nonpyogenic	1	6
I63.8	Other causes	1	228
I63.9	Unspecified cause	50	2122

Figure S1 A and B. Unadjusted cumulative incidence curves for ischemic stroke due to A) embolism of cerebral arteries and B) other causes.

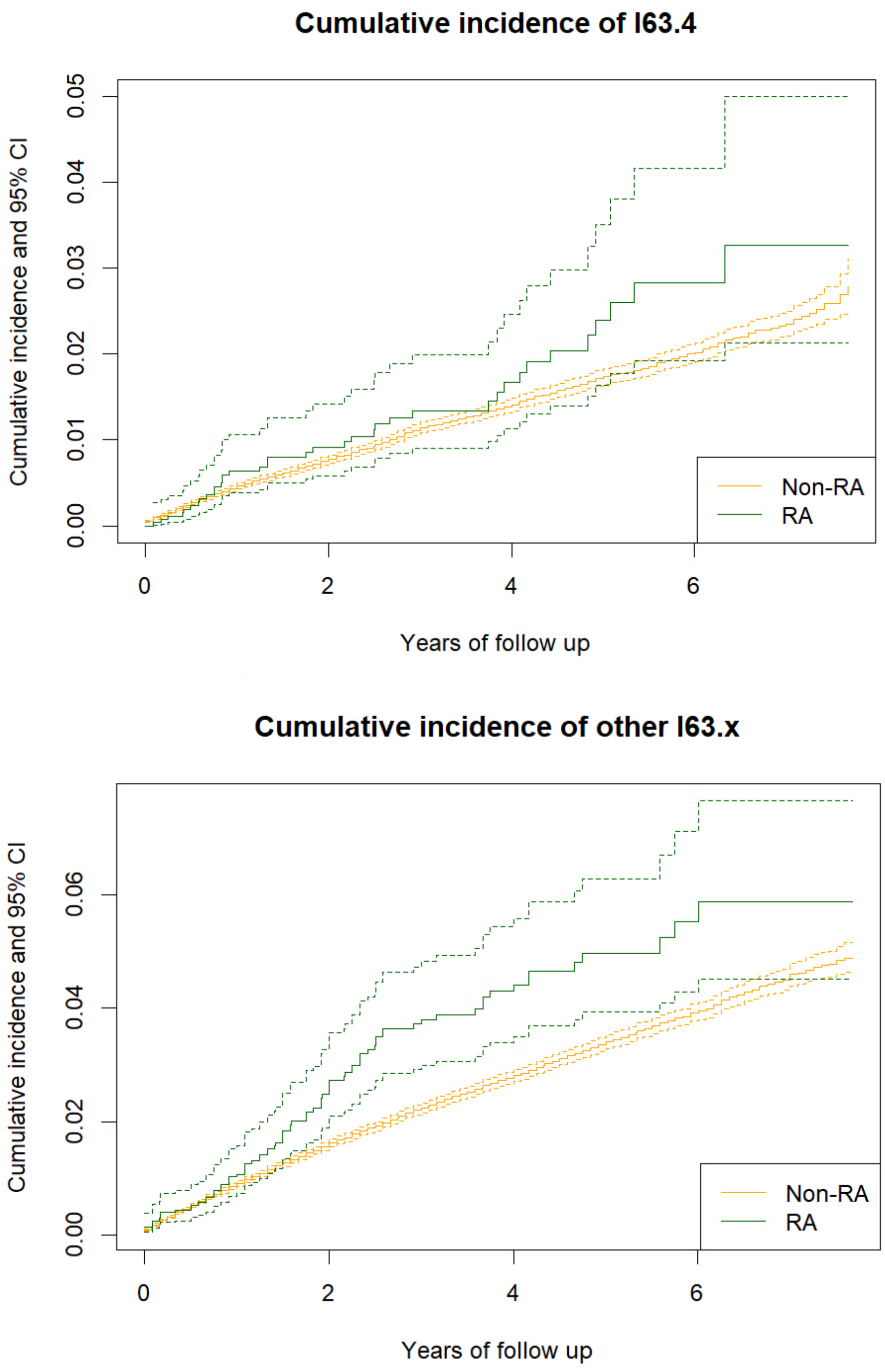

Figure S2. Unadjusted cumulative incidence curves for ischemic stroke in subgroups (A-H).

A: Age at first AF diagnosis ≤ 73 (N: RA=1168, non-RA=82 634).
B: Age at first AF diagnosis >73 (N: RA=1582, non-RA= 76 236).
C: Diabetes (N: RA=444, non-RA= 20 827).
D: No diabetes (N: RA= 2306, non-RA= 138 052).
E: Hypertension (N: RA=1894, non-RA=: 94 990).
F: No hypertension (N: RA=858, non-RA= 63 889).
G: Atherosclerotic CVD (N: RA=897, non-RA=41 257).
H: No atherosclerotic CVD (N: RA= 1853, non-RA=117 622).

A. Age at first AF diagnosis ≤ 73

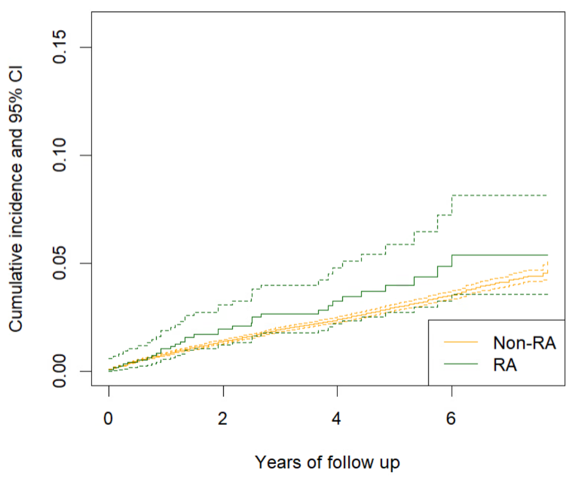

B. Age at first AF diagnosis >73

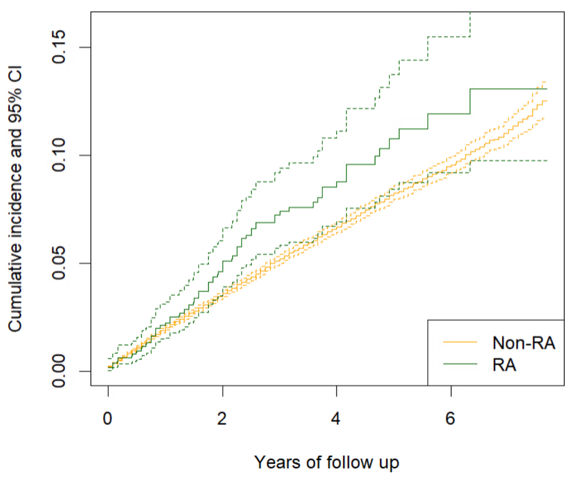

C. Diabetes

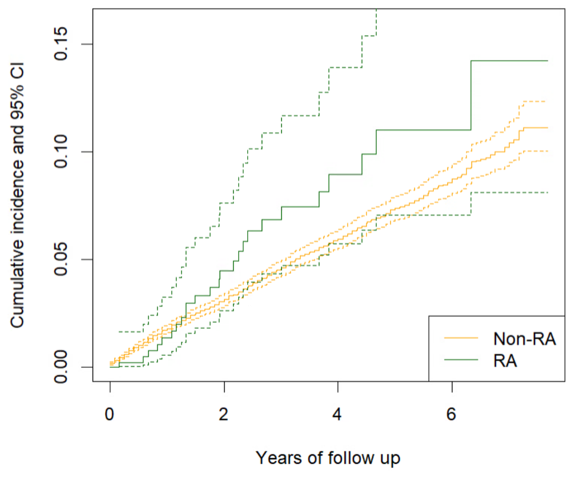

D. No diabetes

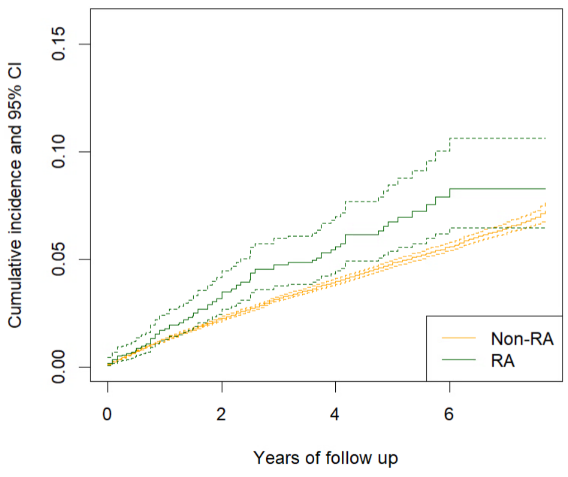

E. Hypertension

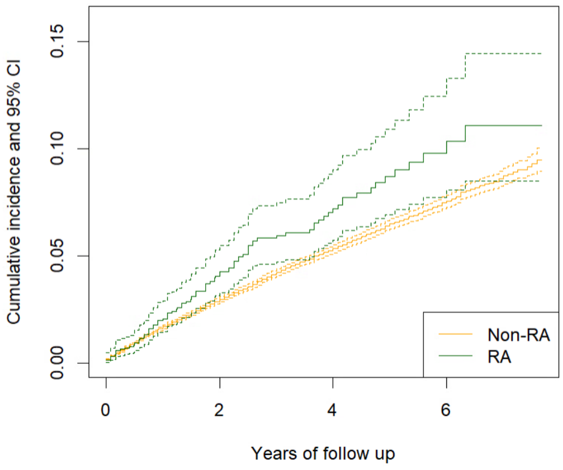

F. No hypertension

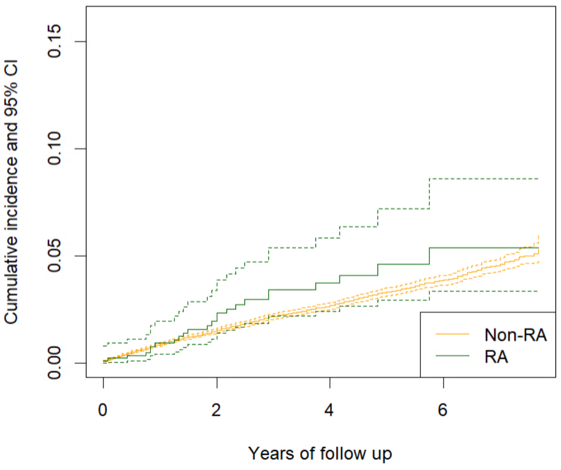

G. Atherosclerotic CVD

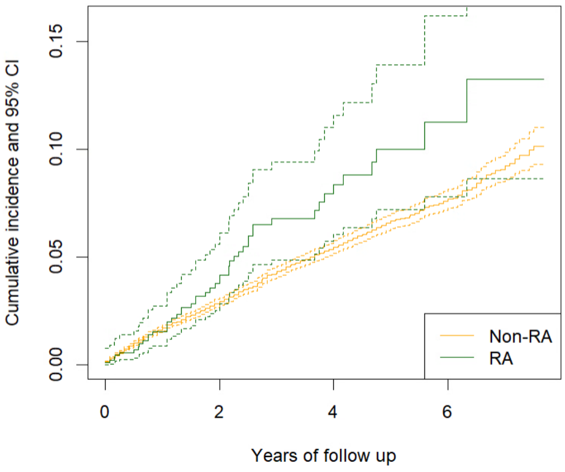

H. No atherosclerotic CVD

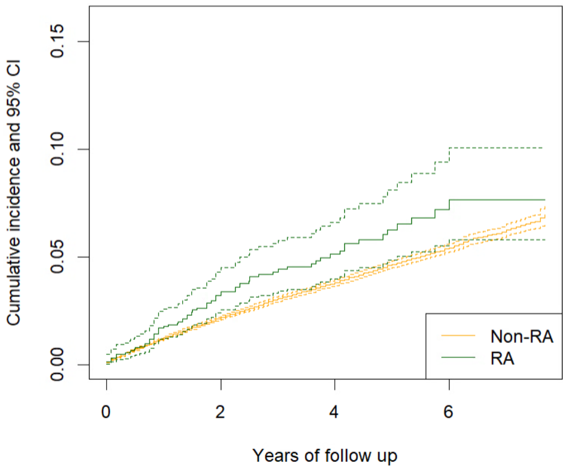

Supplement: keae458_Supplementary_Data [file keae458_supplementary_data.pdf]
